# Machine-learning-guided recognition of α and β cells from label-free infrared micrographs of living human islets of Langerhans

**DOI:** 10.1038/s41598-024-65161-7

**Published:** 2024-06-20

**Authors:** Fabio Azzarello, Francesco Carli, Valentina De Lorenzi, Marta Tesi, Piero Marchetti, Fabio Beltram, Francesco Raimondi, Francesco Cardarelli

**Affiliations:** 1 NEST Laboratory, Scuola Normale Superiore, Pisa, Italy; 2 Laboratorio di Biologia Bio@SNS, Scuola Normale Superiore, Pisa, Italy; 3 Department of Clinical and Experimental Medicine, Islet Cell Laboratory, University of Pisa, Pisa, Italy

**Keywords:** Computational biology and bioinformatics, Data acquisition, Data processing, Functional clustering, Image processing, Machine learning, Biophysics, Biological fluorescence

## Abstract

Human islets of Langerhans are composed mostly of glucagon-secreting α cells and insulin-secreting β cells closely intermingled one another. Current methods for identifying α and β cells involve either fixing islets and using immunostaining or disaggregating islets and employing flow cytometry for classifying α and β cells based on their size and autofluorescence. Neither approach, however, allows investigating the dynamic behavior of α and β cells in a living and intact islet. To tackle this issue, we present a machine-learning-based strategy for identification α and β cells in label-free infrared micrographs of living human islets without immunostaining. Intrinsic autofluorescence is stimulated by infrared light and collected both in intensity and lifetime in the visible range, dominated by NAD(P)H and lipofuscin signals. Descriptive parameters are derived from micrographs for ~ 10^3^ cells. These parameters are used as input for a boosted decision-tree model (XGBoost) pre-trained with immunofluorescence-derived cell-type information. The model displays an optimized-metrics performance of 0.86 (i.e. area under a ROC curve), with an associated precision of 0.94 for the recognition of β cells and 0.75 for α cells. This tool promises to enable longitudinal studies on the dynamic behavior of individual cell types at single-cell resolution within the intact tissue.

## Introduction

In his 1869 doctoral thesis, the German physician and pathologist Paul Langerhans reported the microscopic observation of dispersed small-cell clusters amidst the acinar glandular cells in the pancreas of rabbits^1^. These aggregates of cells, now known as ‘islets of Langerhans’, are regarded as key mini-organs responsible for finely regulating blood glucose homeostasis (and its mis-regulation in diabetes), nutrient sensing, and other related metabolic functions^2,3^. Human islets comprise five main types of hormone-secreting endocrine cells (i.e. α-, β-, δ-, ε- and PP-cells)^3^, of which α and β cells are by far the most abundant (> 90%)^3^ and studied. Indeed, α and β cells secrete the two primary hormones, glucagon and insulin respectively, which regulate blood-glucose levels^4^. Undoubtedly, the ability to directly study their behavior in living islets would be essential for gaining a comprehensive understanding of glucose homeostasis in health and disease^5–10^. Historically, investigations have been hampered by two main technical challenges: (i) the anatomical location and sparse distribution of islets in pancreatic tissue^2^, and (ii) the need to identify α and β cells within the islet. At present, the former challenge is bypassed by extracting islets from the pancreatic tissue and maintaining them alive for further investigations or the use of pancreatic slices^11^. Building on this, the transplantation of living islets into the anterior chamber of a mouse eye was recently proposed as a strategy to investigate islet biology non-invasively and longitudinally under pathophysiologically relevant conditions^12,13^. By contrast, the latter challenge, i.e. the successful identification of α and β cells within the intact islet, remains mostly unattained. For instance, in the studies conducted so far on mouse intact islets, the measured cellular responses/parameters were arbitrarily attributed to β-cells in light of their predominance in the islet (65–80%^3^), effectively forgoing to consider the fractional contribution of α cells^14,15^. More recently, Wang and collaborators measured the metabolic response to glucose stimulation in living mouse islets and set out to discriminate the differential response of α and β cells relying on the assumption that α cells are predominantly located in the periphery and β cells in the core of the mouse islet^16^, as widely accepted based on immunohistochemistry data^16,17^. Unfortunately, this assumption does not hold true for human-derived islets, primarily because of the characteristic intermingled distribution of α and β cells in the human model, which favors heterologous contacts between the two cell types^2^. Worthy of note, in a pioneering work by Rouiller and co-workers in 1990^18^, it was shown that α and β cells disaggregated by mild trypsinization from freshly-isolated rat islets could be distinguished by fluorescence activated cell sorting (FACS) mostly on the basis of their intrinsic autofluorescence (due to flavoproteins elicited at 488 nm). Building on this knowledge, here we implemented a machine-learning-based approach for the recognition of α and β cells directly from label-free infrared micrographs of living and intact human Langerhans islets. It exploits the label-free microscopy dataset recently generated by some of us in the effort to study the metabolic response of human islets to glucose stimulation^19^. Data consist of autofluorescence measurements and identity of 312 α cells and 654 β cells collectively from 15 human islets, obtained from 4 healthy donors. Islets autofluorescence was stimulated at 740 nm by multiphoton excitation and measured both in intensity and lifetime in the 420–460-nm optical window, which is dominated by NAD(P)H emission and lipofuscin signals^20,21^, while cell identity (α or β) was obtained upon tissue fixation and immunohistochemistry against glucagon and insulin. Here a number of features (i.e. 151) able to parametrize the autofluorescence of the islet at the single-cell level are extracted and used as input for a boosted decision-tree model (XGBoost) trained with the immunofluorescence-derived cell-type information. The model displays an optimized-metrics testing performance of 0.86 (area under a ROC curve), with an associated precision of 0.94 for the recognition of β cells and 0.75 for α cells. This machine learning tool allows α and β cell recognition in intact islets without need to perform immunostaining, holding the potential to enhance conventional imaging on human islets, thus enabling longitudinal studies on the behavior of single cells (and cell populations based on their type) within intact tissue in both physiological and pathological contexts.

## Results and discussion

### From image collection to dataset creation

**Figure 1:**
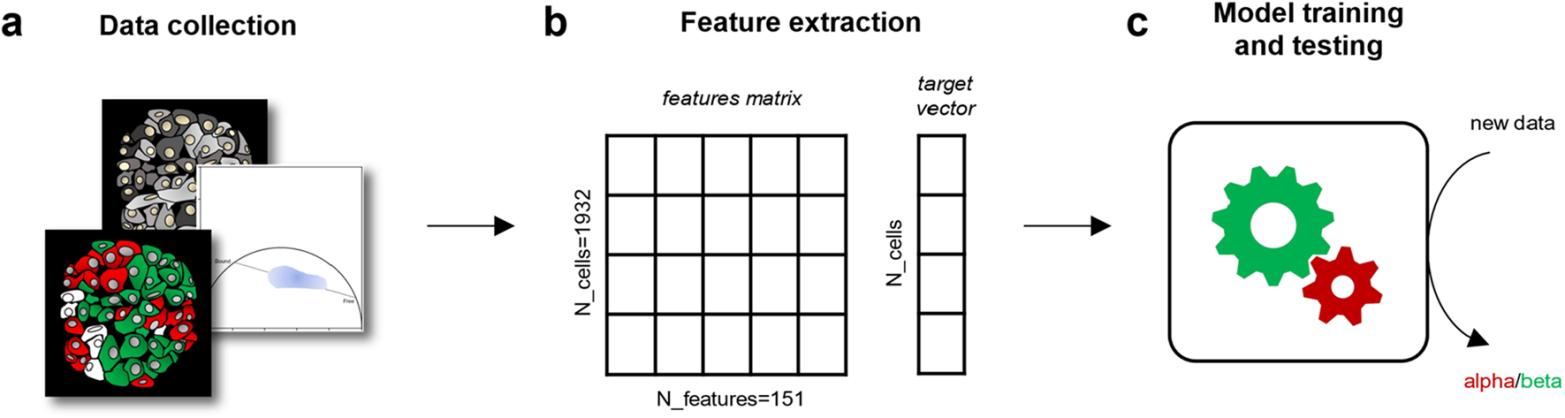
General workflow. (**a**) Using a fluorescence microscope equipped with a FLIM (Fluorescence Lifetime Imaging Microscopy) module, data were collected from 15 Human Langerhans islets in three types of images: autofluorescence intensity (cartoon in grayscale), FLIM images, typically visualized as a phasor plot (blue cloud), and immunofluorescence images (red and green cartoon, where red represents α cells, and green represents β cells). (**b**) Single-cell data were obtained through manual segmentation of the acquired images, which resulted in one image per each segmented cell. For each cell, a number of parameters were calculated and included in a dataset then used to train a Machine Learning algorithm. (**c**) After a testing phase, the model can be employed to determine cell identity from new images without the need for additional immunostaining.

The whole machine learning workflow is schematically represented in Fig. 1. In brief, it starts with an algorithms training which consists of three main phases, namely: (i) live-islet autofluorescence intensity imaging by exciting at 740 nm and collecting in the 420–460-nm range, which is dominated by NAD(P)H and lipofuscin signals; (ii) NAD(P)H auto-fluorescence lifetime imaging at the same focal plane in live islets at both low (2.2 mM) and high glucose (16.7 mM), with subtraction of the lipofuscin intrinsic signal, to produce metabolic data in terms of balance between free and protein-bound NAD(P)H; (iii) islet fixation and immunostaining using antibodies against glucagon and insulin to identify single α and β cells and then extract single-cell information from both intensity and lifetime data through spatial matching of immunofluorescence and live-islet acquisitions (Fig. 1a). At this point, we curate the manual processing of experimental data to extract a set of numerical features (Fig. 1b) and store them in a feature matrix. Each row of the matrix is associated with an outcome (cell identity, obtained by immunofluorescence) denoted as either ‘α’ or ‘β’, and this is described in the target vector. At this point, the majority of the dataset is used to train a model that captures the relationship between numerical features and cell type (Fig. 1c). The rest of dataset is used during the testing phase, where the performance of the model is evaluated by predicting cell type using the data portion withheld from the training phase. Upon successful completion of the testing phase, the model becomes capable of inferring cell type (i.e. the target vector) from newly collected data of sole autofluorescence and lifetime imaging, eliminating the need of performing immunostaining for cell type recognition.

**Figure 2:**
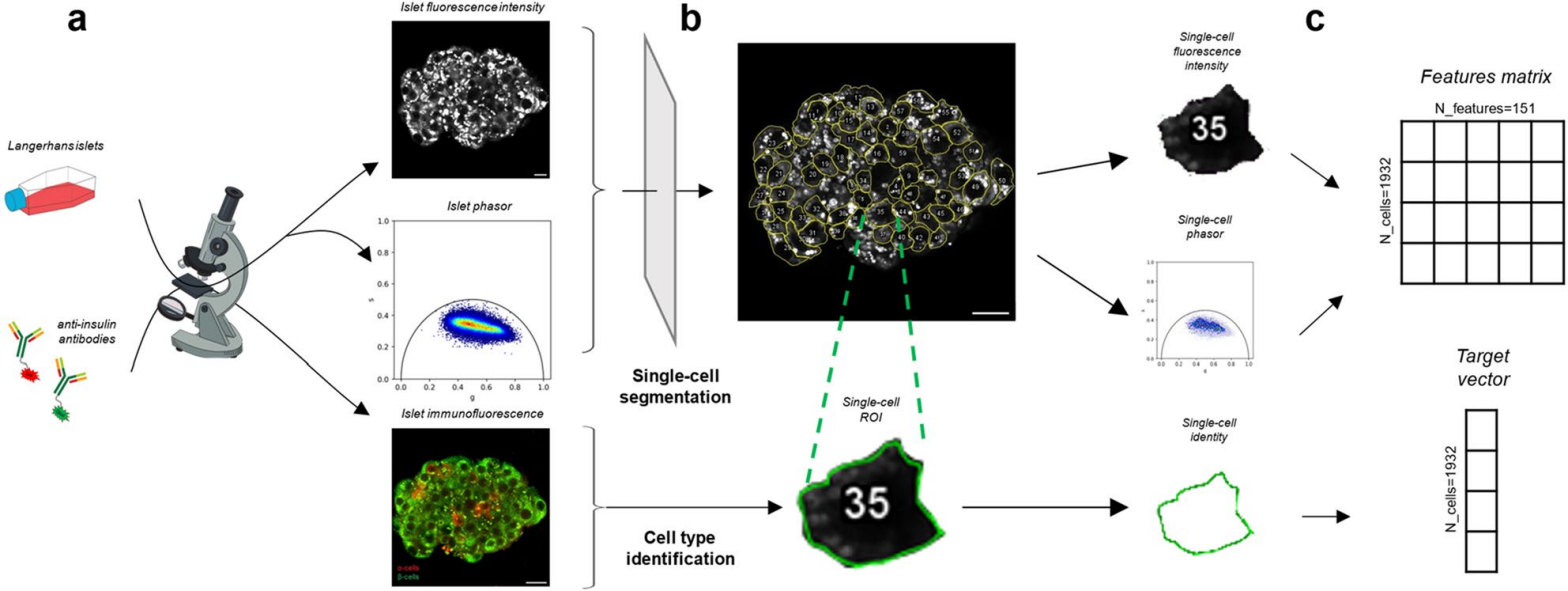
From image collection to dataset creation. (**a**) Human Langerhans islets' autofluorescence and lifetime are measured using label-free fluorescence microscopy, giving an autofluorescence image (top) and a phasor plot (center) of the islet as result of the live-cell imaging step. In the following step, fixation and permeabilization are performed. Then, islets are incubated with antibodies (green: anti-insulin, red: anti-glucagon), leading to the corresponding immunofluorescence image (bottom) of the islet. (**b**) Already obtained autofluorescence images are manually segmented by outlining Regions Of Interest (ROIs), obtaining single-cell data. Likewise the entire islet, each single cell has an associated autofluorescence image, a phasor plot, and cell identity information obtained from immunofluorescence. (**c**) Single-cell images are used to extract 151 features per cell, which are organized in a feature matrix. In this matrix, each row represents a single cell and each column corresponds to a specific feature. The target vector contains information about cell identity, which are derived from the immunofluorescence images.

In more detail, to build the input dataset we performed label-free multi-photon imaging of human islets (Fig. 2a), which provided two distinct types of data: islets autofluorescence intensity (Fig. 2a, top panel) and lifetime (Fig. 2a, center panel) micrographs. The autofluorescence signal was elicited at 740 nm through multiphoton excitation and collected in the 420–460-nm optical window. Each islet was measured twice: first, at 2-mM glucose concentration, which maintains a starvation condition, and then after 5–10 min exposure to 16-mM glucose concentration, which stimulates insulin secretion from β cells. Following multiphoton imaging of live islets, these were fixed and prepared for immunofluorescence (Fig. 2a, bottom panel). This step involves tissue fixation, followed by permeabilization and, ultimately, incubation with anti-glucagon (red signal) and anti-insulin (green signal) antibodies to identify α and β cells, respectively. After image acquisition, manual segmentation (Fig. 2b) was carried out to extract single-cell information: 151 features were extracted (Fig. 2c) and used to construct what is referred to as the ‘feature matrix’. Each row of the matrix is associated with an outcome, specifically cell identity, denoted as either ‘α’ or ‘β’, and this is described in the ‘target vector’. At the end, the feature matrix contains data from N = 1932 cells, with each cell associated with N = 151 features. In contrast, the target vector exclusively contains immunofluorescence-derived information on cell identity.

**Figure 3:**
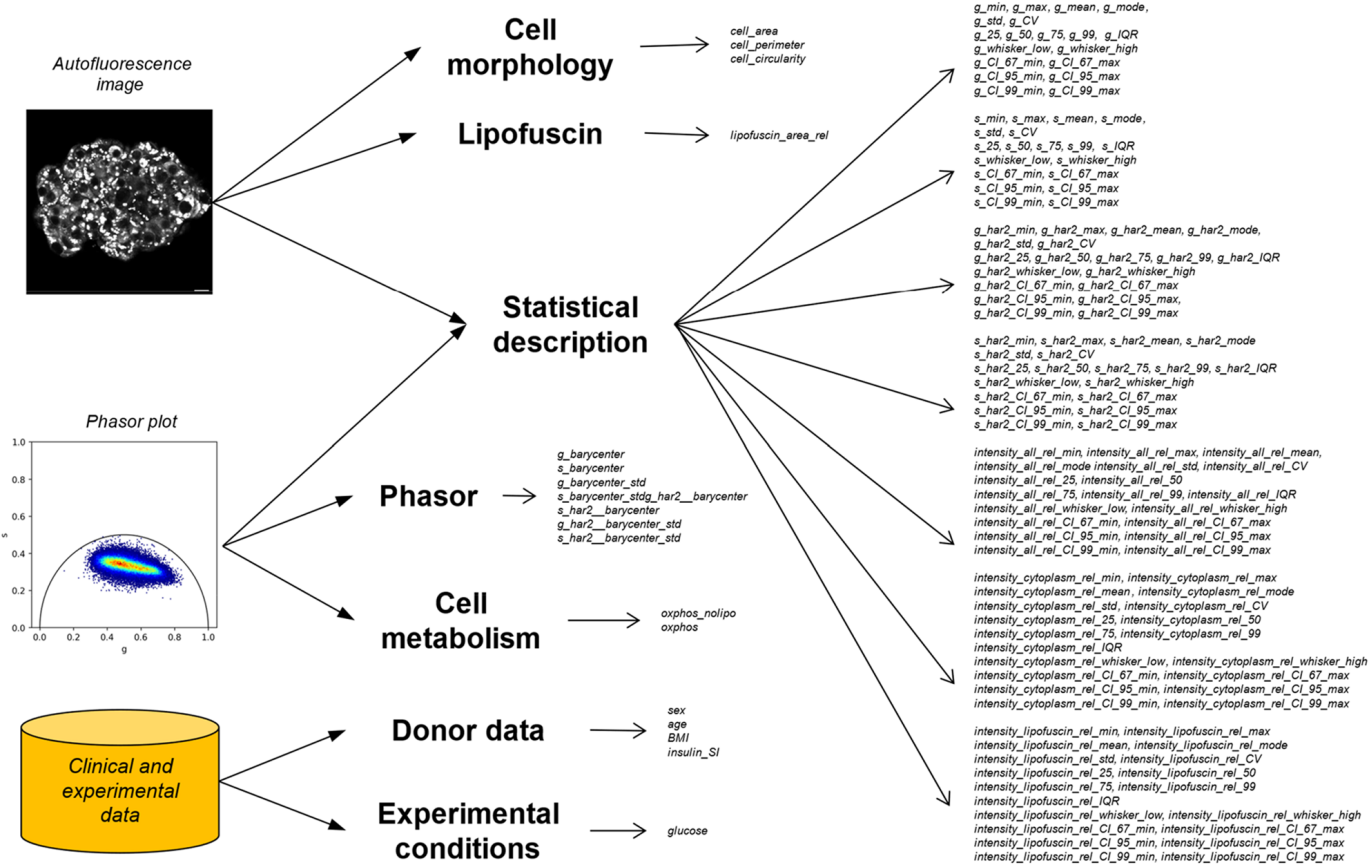
Overview of calculated features. In total, 151 features (in italic) have been extracted from phasor, autofluorescence, clinical, and experimental data. These features describe or summarize (in bold): Phasor plot characteristics, Cell metabolism, Cell morphology Lipofuscin content, Donor-related demographic and clinical data, Experimental conditions. In addition, various descriptive statistics parameters are used as general-purpose descriptors to summarize both autofluorescence and phasor relevant characteristics.

Most of the numerical entries of the feature matrix (the complete list is reported in Supplementary Material) are derived from either autofluorescence intensity and lifetime data through the utilization of descriptive statistics parameters including, for instance, minimum and maximum values, trends, range of most common values, and data dispersion (Fig. 3). Notably, in the optical window used for NAD(P)H detection, human islets also contain marked autofluorescence originating from lipofuscin-enriched granules^20,21^. These granules, byproducts of lysosomal digestion, are primarily composed of lipids and proteins, and directly correlate with age of donor^19,22^. Since α and β cells are known to possess different amounts of lipofuscin^19^, we decided to include a parametrization of lipofuscin granules by estimating their area normalized by the cell area. Cell morphology is instead described by three key parameters: cell area, perimeter, and circularity. Circularity quantifies how closely the cell shape resembles a perfect circle, with a value of 1 indicating a perfect circle. For what concerns autofluorescence lifetime data, the Fourier transformation converts the lifetime decay measured in each pixel of the image into a data point in the phasor plot, characterized by three parameters: the ‘g’ and ‘s’ coordinates, which describe the time constant of autofluorescence decay, and the frequency of observation of each specific set of ‘g, s’ coordinates. Phasor clusters were quantitatively analyzed by extracting both the cluster barycenter and its standard deviation. In addition, by combining phasor-FLIM data acquired at two glucose concentrations, additional information about cell metabolism could be obtained: in fact, the shift in NAD(P)H lifetime upon glucose stimulation can be used as a descriptor of the average metabolic balance between glycolysis and oxidative phosphorylation in α and β cells. Finally, infrared-imaging-derived features were supplemented by adding donor-related clinical parameters (Table S1) such as age, body mass index (BMI), and the insulin stimulatory index (SI), this latter intended as the overall insulin secretion efficiency of donor-derived islets measured by a standard ELISA assay.

### Explorative data analysis reveals moderate association of features to α/β-cell type

**Figure 4:**
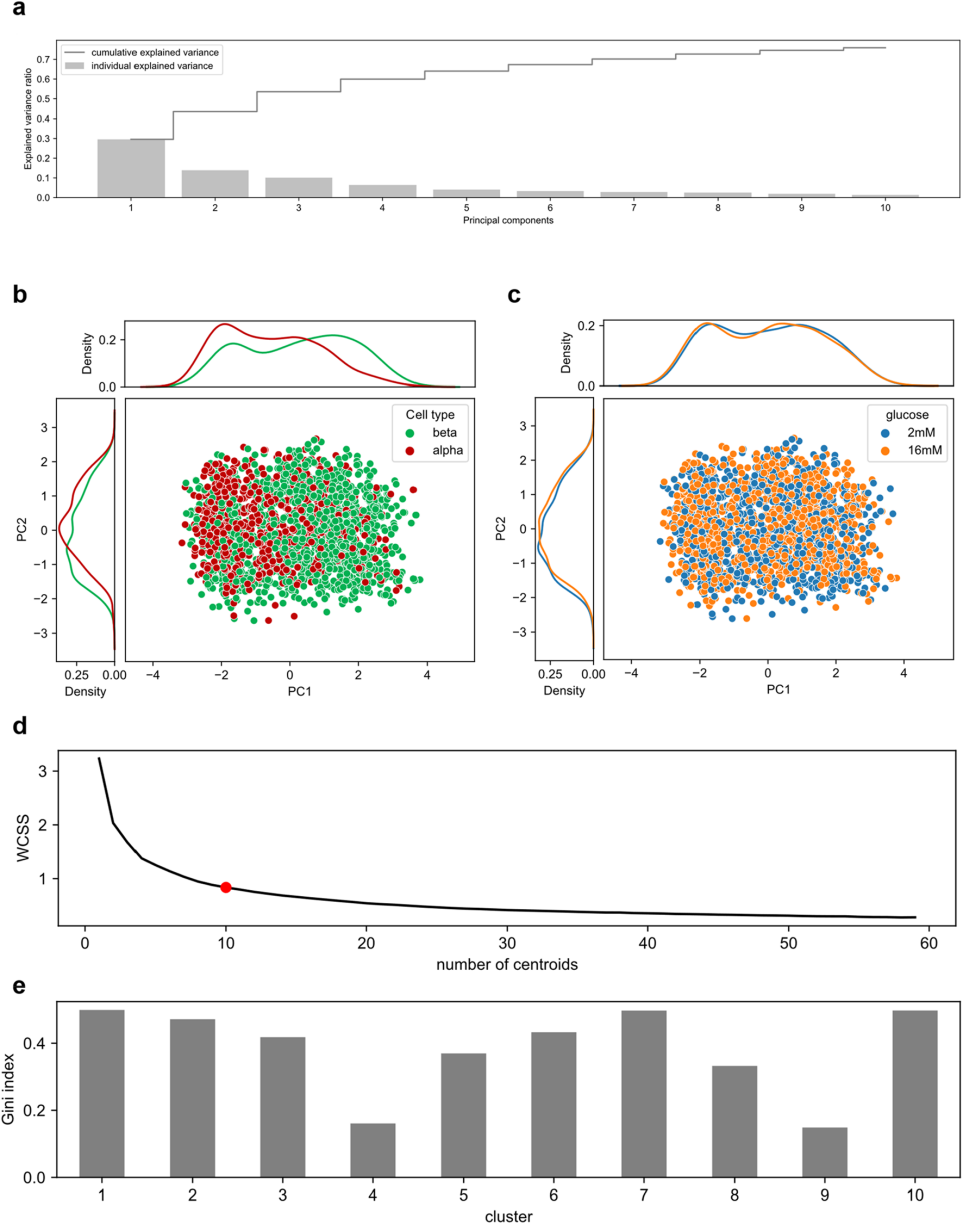
Explorative data analysis with PCA and K-means clustering. The dataset dimensionality has been reduced from 151 to 2 using PCA to allow graphical representation. (**a**) A graphical representation of explained variance respect to the number of principal components gives an idea on how much components/dimensions are needed to retain enough information after dimensionality reduction. The explained variance drops rapidly, meaning that two components are enough to visualize the data without significant information loss. (**b**) The bidimensional PCA scatterplot (bottom, right) appears separated on the basis of cell type, despite mildly clustered. This suggests classification is possible using complex algorithms, maybe using a supervised approach or neural networks, as confirmed their distribution using kernel density estimation plots on the first principal component (top) and second principal component (botton, left). (**c**) Using experimental glucose concentration as colormap, it becomes evident that glucose concentration does not significantly affect cell classification. This implies that glucose concentration has low classification power, implying that the classification model will be able to classify cells independently of this experimental condition. (**d**) The elbow method allowed to choose a suitable number of clusters to have good performance by computing the WCSS (Within-Cluster Sum of Squares, i.e. sum of squared distances of all points from the centroid they belong) indicates for each iteration. The elbow (red dot) indicates the optimal number of clusters, which is 10. (**e**) The Gini impurity index has been computed for all clusters to assess within-cluster heterogeneity. The ideal case would be having only one class per cluster, which would result in Gini = 0. However, the average Gini among all clusters is 0.37.

To facilitate the exploratory data analysis we employed the Principal Component Analysis (PCA)^23^ as a dimensionality reduction algorithm. We first chose the optimal number of components to avoid information loss and plot the explained variance with respect to the number of components (Fig. 4a). The explained variance decreases rapidly even with few components, thus we reduced the dimensionality of the dataset from 151 to 2, making the entire dataset amenable to visualization in a 2D Cartesian plot and enabling us to observe the impact of specific features through color mapping. The PCA outcome is represented as a 1932 × 2 matrix in order to visualize only single-cell data.

For instance, if data are color-coded according to cell type, α and β cells show mild segregation (Fig. 4b, bottom right), as confirmed by kernel density estimation (KDE) plot on both the first principal component (Fig. 4b, top) and second principal one (Fig. 4b, bottom left), suggesting that classification might be reached, but using sophisticated supervised algorithms. If cells are color-coded by means of the glucose concentration used in the experiment (Fig. 4c), it becomes challenging to accurately distinguish between α and β cells. This implies that glucose concentration may not possess strong classification power, thus the algorithm might be able to classify cells independently of the experimental glucose concentration used. To support this hypothesis more quantitatively the need of a Supervised Learning approach, we conducted a clustering analysis using the widely-employed k-means algorithm. First, we selected the proper amount of clusters using the elbow method. This consists in performing k-means iteratively by progressively increasing the number of clusters and calculating, for each iteration, the WCSS (Within-Cluster Sum of Squares), which represents a quantitative evaluation of how much data points are tight-bound to the cluster centroid. The optimal number of clusters should ideally match the number of classes of the classification problem (i.e. 2), but this would perform poorly here, as demonstrated by the elbow-test results (Fig. 4d). The best score is reached for the highest number of clusters, but this in turn is a sign of data overfitting: the suitable number of clusters chosen was 10 (Fig. 4d, red dot). For the chosen number of clusters, we assessed the performance of k-means by quantifying data heterogeneity within each cluster using the Gini impurity index (Fig. 4e), exploiting the labels on the data obtained by immunofluorescence. The ideal scenario would be Gini = 0, which indicates that the cluster only contains one class. Other way round, if Gini = 1 (worst case), it means that data within the cluster is entirely diverse. The average Gini coefficient across all clusters is 0.37, which confirms our hypothesis about the supervised approach. To give the reader a more synthetic view of the results, we calculated the ROC_AUC (i.e. area under a ROC curve) of a two-component K-Means on PCA data, obtaining 0.60, thus reinforcing our conclusions: the explorative data analysis using PCA showed mild clustering of α and β cells, prompting us to use supervised classification algorithms.

### Supervised learning of an accurate α/β-cell discriminator

**Figure 5:**
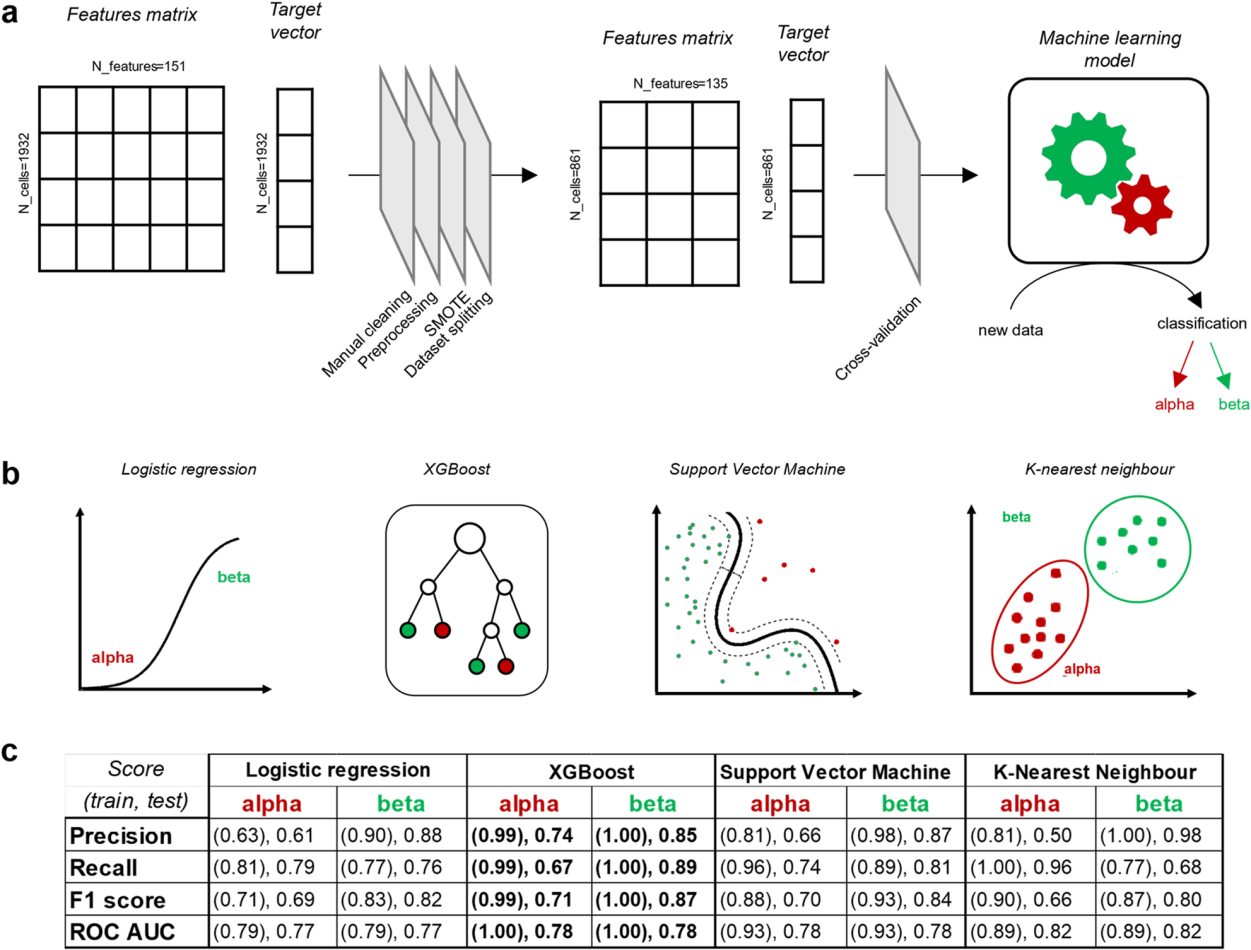
Supervised-learning results from four different models. (**a**) After creating the feature matrix and target vector, data undergo several preprocessing steps to enhance the performance and stability of classification. The process starts with manual cleaning, where only cells with clearly defined identities are retained in the dataset, excluding over a thousand cells, resulting in a cleaned dataset with 861 cells. Preprocessing includes encoding categorical features, handling missing values, handling outliers, and scaling the data. The dataset is then rebalanced using SMOTE (Synthetic Minority Oversampling Technique), and it is split into training and test sets. The training set, after SMOTE, comprises 970 cells and 151 features. Before training, cross-validation and hyperparameter tuning are performed to obtain a stable and high score. The model is tested on the testing data, which can be considered as new, unseen data. The original data is cleaned to improve algorithm performance. (**b**) Four different algorithms are tested and compared: multivariate logistic regression, boosted decision tree (XGBoost), Support Vector Machine for classification, and K-Nearest Neighbor for binary classification. Each algorithm is optimized using the most common hyperparameter range and Grid Search as the optimization algorithm. (**c**) Evaluation of precision, recall, F1 score, and the area under an ROC curve reveals that XGBoost is the most promising algorithm in terms of classification performance and stability. XGBoost is further optimized with Optuna, allowing for the selection of a wider hyperparameters range to improve its performance.

Before training the model, we cleaned the dataset by manually reviewing cells, and we discarded those for which cell identity could not be confidently determined to prevent the introduction of noise into the training phase (Fig. 5a). The following step involved data-preprocessing operations to favor model performance and stability: these included numerical encoding categorical features, features scaling, handling of missing values and outliers. A critical point in data preprocessing was that of addressing dataset imbalance, i.e. the unequal number of α and β cells in the training set. Neglecting cells from the most abundant class (i.e. β cells) could lead to a biased model due to the high biological heterogeneity of Langerhans islets (Table S2)^24^, considering that several algorithms are built on the hypothesis of balanced classes as inputs). To address this, we employed the Synthetic Minority Oversampling Technique (SMOTE)^25^. This algorithm leverages existing data to generate synthetic data entries, rebalancing the β:α ratio of the whole dataset from 2:1 to 1:1, thus improving model training. At this point, the dataset was divided into the ‘training’ and ‘test’ sets (Fig. S1) to prevent overestimation of model performance during testing. Model performance and stability were further enhanced by implementing both Cross Validation and hyperparameters tuning procedures. Repeated stratified fivefold Cross Validation (with 3 repetitions) was applied, and Grid Search was chosen for cross-validation and hyperparameters tuning. The area under a ROC curve (ROC_AUC) was selected as the optimization metric, given its appropriateness for machine-learning problems based on imbalanced classes, as in this case. Four different algorithms were trained and tested (Fig. 5b) using 970 cells for training (a mix of real data and synthetic data generated by SMOTE) and 216 cells (real data) for testing. Training and testing performances were then compared based on various metrics, including precision, recall, and F1 score, in addition to the area under the ROC curve. Regarding the two cell types under study (Fig. 5c), β cells generally exhibited scores exceeding 0.80, while α cells exhibited slightly lower overall performances ranging from 0.60 to 0.70. This discrepancy may be linked to the degree of cell-type-specific information embedded in the extracted biological features. For instance, it was recently demonstrated and confirmed that β cells have a significantly higher lipofuscin content compared to α cells (i.e., twofold)^22^ and display a distinct metabolic shift toward oxidative phosphorylation upon glucose stimulation^26^, which is not as clearly observed in α cells^19^. In this scenario, the extracted features convey the proper amount of information to explain the behavior of β cells with confidence, while it takes more effort to take decisions on α cells. All the tested algorithms showed high performance, but unsatisfactory precision or recall on α-cell classification, with the exception of XGBoost. XGBoost displayed high performance and classification stability (i.e. all the computed scores were quite similar within the same class), and was thus selected for a further optimization step.

**Figure 6:**
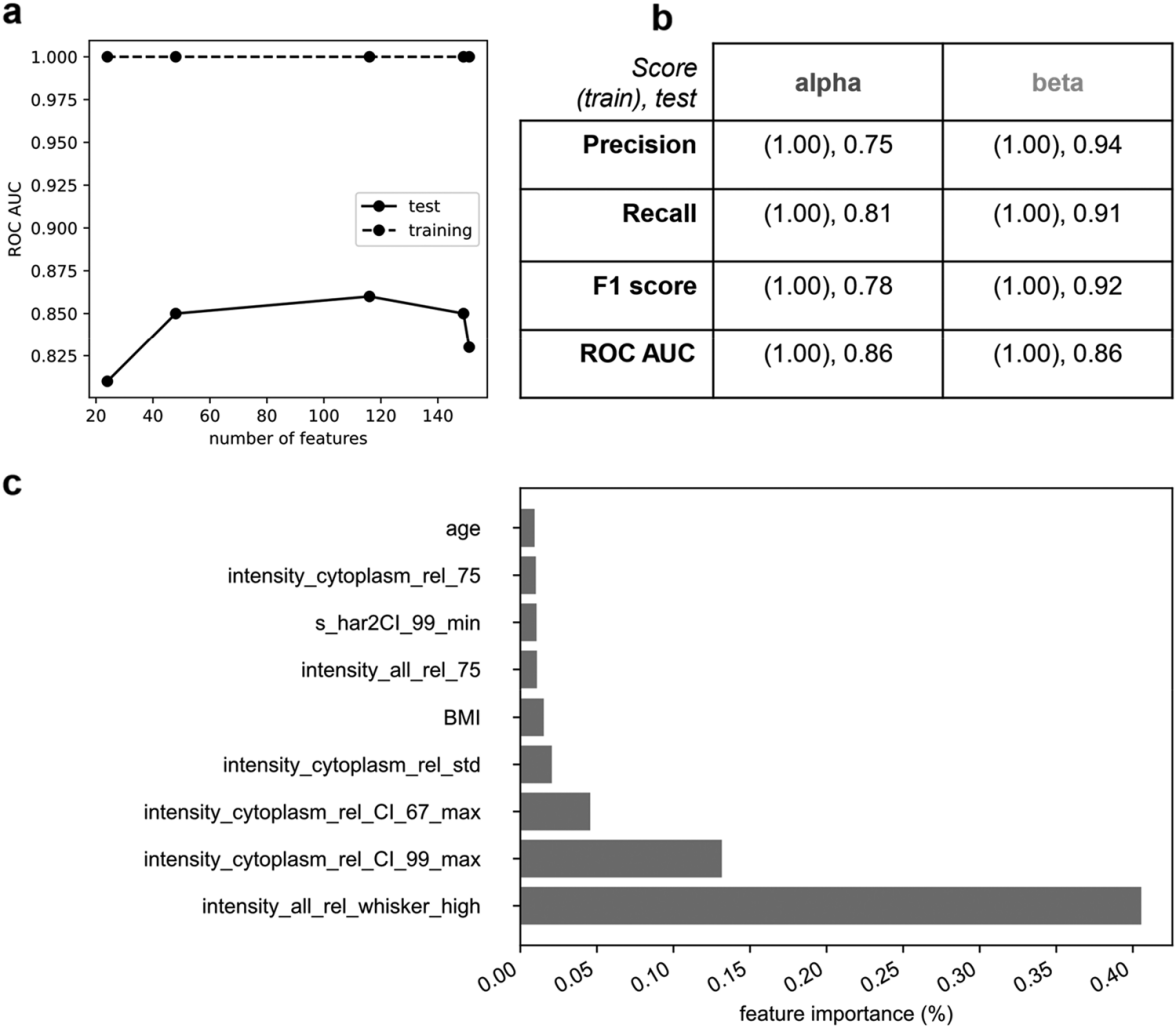
XGBoost Optimization with Optuna, Feature Importance, and Model Stability Assessment. XGBoost performances have been further improved via feature selection and larger hyperparameters tuning using Optuna. (**a**) We selected the optimal number of features by training XGBoost with Optuna, selecting a subset of the most important features, discovering that almost all features are needed for optimal performance. (**b**) The best model has been obtained for 116 features; it shows a ROC_AUC = 0.86, and precision comparable with FACS on dissociated cells made by other researchers. (**c**) By plotting the 9 best features, we can observe that more than 50% of classification power comes almost entirely from autofluorescence images.

The optimization of XGBoost was performed by using Optuna^27^ that, contrary to Grid Search, does not evaluate all possible hyperparameter combinations but efficiently explores the hyperparameter space through sampling and pruning algorithms. For feature selection, we leveraged the embedded method of the XGBoost algorithm, which provides an importance score for each feature ranging from 0 to 1, based on their significance within the classification task. After an initial XGBoost training using all features, these were sorted from the most relevant to the least, and a new training phase initiated with a restricted number of features and setting different cutoff thresholds (Fig. 6a). This process was aimed at enhancing model performance and, potentially, at reducing computational cost. A detailed view of all computed scores can be found in Table S3. The model with the highest performance achieved a ROC_AUC of 0.86 by using the top 116 features out of 151, thus indicating that the majority of the features are essential for optimal classification (Fig. 6b). This is likely due to the high biological heterogeneity of Langerhans islet cells, both within and across donors. As mentioned earlier, Rouiller and co-workers showed that α and β cells disaggregated from rat islets can be separated using fluorescence-activated cell sorting (FACS). This separation relied on their intrinsic autofluorescence (mostly due to flavoproteins elicited at 488 nm) and the characteristic size of the cells^18^. This observation prompts us to consider the significance of delving deeper into the analysis of intrinsic signals (e.g. by building a more complex algorithm as deep learning, at expense of interpretability) or by extracting more information-rich features to achieve similar or higher model performances based on standard imaging. However, a classification algorithm is needed to not underperform α- or β- cells classification, as evidenced by the K-Means analysis in the Explorative Data Analysis. In order to make a direct comparison with XGBoost, we applied the same pre-processing to the dataset as in the algorithm training phase, then we applied the 2-component k-Means, obtaining ROC_AUC = 0.72, much lower than XGBoost (Fig. 6b). Coming back to model interpretability, XGBoost has an embedded method which allows to extract and identify the most important features able to explain the classification power. By plotting the nine most important features (Fig. 6c) we can observe that 6 out of 9 features are related to static autofluorescence, and the first three are able to explain more than 60% of the classification power, suggesting that most of classificatory information is encoded in the autofluorescence intensity. Indeed, by color-mapping the PCA plot (Fig. S2) for the most important feature (i.e. “intensity_all_whisker_high”), it can be seen that it follows the cell-type distribution shown in KDE plots. This observation is also corroborated by previous ones on the higher lipofuscin content of β cells^22^ and their increased fluorescence intensity due to oxidative metabolism^28–30^ as compared to α cells. To ensure model stability, we conducted additional assessments. First, we increased the number of folds from 5 to 10, implementing tenfold repeated stratified cross-validation. All training and Optuna-optimization steps were repeated and the same evaluation scores calculated (Table S4a), showing ROC_AUC = 0.86, which is comparable to the fivefold cross-validation results (Fig. 6b) together with the other metrics. Additionally, we performed the ‘Salzberg test’^31^, a method that involves shuffling the labels in the target vector of the training dataset, allowing the algorithm to learn from noise. This test showed a ROC_AUC = 0.53, which is a 33% decrease for both training and testing (Table S4b), confirming that the model optimized during the standard training procedure was not influenced by overfitting. Furthermore, we attempted to classify data that had been excluded from training during the dataset cleaning procedure. The resulting ROC_AUC was 0.64, and all computed metrics displayed lower performance (Table S4c), thus validating the effectiveness of the cleaning procedure.

## Conclusions

In the present work we have implemented a boosted decision-tree model (XGBoost) designed to identify α and β cells in label-free optical-microscopy images of living human pancreatic islets without need to perform tissue fixation and immunostaining. The obtained performance metrics (i.e. area under a ROC curve) is 0.86, with a precision of 0.94 for the recognition of β cells and 0.75 for α cells. As compared to the previous results obtained by Rouiller and co-workers in 1990^18^ the present approach marks a decisive advancement in the field because, contrary to FACS, the strategy proposed here does not rely on disaggregated cells, but preserves islet cyto-architecture and, thanks to this, all the possible inter-cellular communication mechanisms, i.e. ‘*paracrine*’ effects. As a direct consequence, the present approach opens to longitudinal studies on the behavior of individual cell types (and single cells) in living islets in both physiological and pathological contexts. Finally, it is worth mentioning that the α/β-cell discriminator implemented here performs well in the human islet, i.e. in a tissue context in which confounding factors such as the presence of lipofuscin granules or the intermingled distribution of α and β cells had previously hindered efforts in cell-type classification.

A few directions for further development of this approach can be envisioned. In this implementation, for example, the algorithm currently relies also on autofluorescence-lifetime data that may not be accessible in more standard instrumentation set ups. Our future plans involve developing an algorithm specifically designed to achieve effective discrimination between α and β cells solely through the utilization of static autofluorescence imaging. Additionally, it is crucial to acknowledge that potential errors may arise in the analysis due to the presence of additional types of cells in the islet, e.g. δ-, PP, ε-cells. The exploitation of specific antibodies against these cells, however, is possible and would pave the way to the successful training of a multiclass algorithm. The introduction of automatic cell segmentation into the procedure would also be highly desirable, and any possible segmentation tool available can be used in synergy with the proposed classification model. Postić and colleagues have developed one, used on mice β-cells^32^, but many general-purpose tools are present, even based on very complex models as neural networks^33^. Segmentation is an actively researched target in the field of image processing and machine learning, available methods still face challenges in terms of reliability and versatility^13,34^, and there is currently no tool specifically able to target human Langerhans islets criticalities as tight cell packing and lipofuscin presence. Furthermore, in the present study, the algorithm was trained mostly on aged healthy donors (Table S1): future developments will have to include extending the set of donors to train the model and classify α/β cells, for instance, in islets from younger donors. Lastly, this approach might help to better characterize α and β cells functional heterogeneity under stressing conditions, such as gluco- and/or lipotoxic stress^35–37^ and pro-inflammatory stress^38,39^, as well as in type 1 and type 2 diabetes^6,40,41^.

## Materials and methods

### Human islet isolation and culture

Data analyzed in the present study derive from four human islet preparations^19^. The features of the non-diabetic organ donors regarding age, sex and body mass index were as follows: #1, 85, M, 27.7; #2, 80, M, 23.03; #3, 46, M, 23.67; #4, 79, M, 26.81. Additional information are reported in Table S1. The procedures were approved by the Ethics Committee of the University of Pisa (21st of November, 2013, #2615). The islets were isolated before the 1st of November 2021.

### FLIM image collection and single-cell segmentation

Before imaging, islets were immobilized in 1% agar hydrogel at low glucose concentration (2.2 mM) in SAB buffer. Two-photon imaging was performed using an Olympus FVMPE-RS microscope equipped with a FLIMbox system (ISS, Urbana Champaign) for lifetime data acquisition. NAD(P)H was excited at 740 nm with 80-MHz repetition rate and autofluorescence collected in 420–460 nm range. Finally, islets were stimulated with glucose to reach a final concentration of 16.7 mM and imaged again after 3–5 min with the same protocol. After two-photon imaging, islets were fixed using paraformaldehyde and permeabilized with Triton X-100, then immunostaining with anti-insulin and anti-glucagon antibodies was performed. After image collection, single cells were manually segmented using Fiji software. After single-cell segmentation, each cell had three associated 512 × 512 matrices: two for ‘g’ and ‘s’ coordinates and one for autofluorescence intensity. This resulted in N = 1932 single-cell images of the same type.

### Feature extraction

Single-cell data, except for immunofluorescence, was stored in R64 files available at figshare (10.6084/m9.figshare.23765169.v1). A Python script was developed to import and extract features from the mentioned matrices. Feature extraction was carried out using the numpy library, and the calculated parameters were stored in a dataset using the Pandas library. Each row of the dataset contains data for donor and single-cell identification, as well as the computed features, resulting in a total of 151 features and 1932 cells. The dataset can be downloaded at 10.6084/m9.figshare.23765169.v1, and we recommend using the specific function implemented for import available at https://github.com/Biofaa/CellTypeClassification.

### K-means clustering analysis

The k-means algorithm was implemented using scikit-learn library (i.e. sklearn.cluster.k_means class). To implement the elbow method, the k-means algorithm was used to iteratively fit the data using the k_means.fit(X) method, where X is the UMAP output (i.e. 1932 × 2 matrix). At each iteration the number of initialized clusters incremented from 2 to 59 and the WCSS (Within-Cluster Sum of Squares) was calculated as:

1 $$WCSS\;\left( {c_{1} ,\;c_{2} , \ldots \;c_{d} } \right) = \frac{1}{N}\mathop \sum \limits_{j = 1 }^{d} \min \left\| {x_{i} - c_{j} } \right\|^{2}$$

where ${\text{c}}_{{\text{j}}}$ is the centroid coordinate of cluster ${\text{C}}_{{\text{j}}}$, ${\text{d}}$ is the number of clusters, $N$ the number of elements ${\text{x}}_{{\text{i}}}$ that belong to the cluster ${\text{C}}_{{\text{j}}}$. After all the WCSS were calculated, the elbow plot was shown (Fig. 4d). As a standard, the optimal number of clusters is found where the so-called elbow of the curve is located (Fig. 4d, *red dot*), approximately at 10 clusters. At this point, the impurity of each cluster has ben assessed by the Gini index:

2 $$G\left( j \right) = 1 - \mathop \sum \limits_{k = 1}^{{N_{k} }} \left( {\frac{{n_{k,j} }}{{n_{j} }}} \right)^{2}$$

where ${\text{N}}_{{\text{k}}}$ is the number of classes of the classification problem (i.e. 2), ${\text{n}}_{{\text{j}}}$ the number of elements of cluster ${\text{C}}_{{\text{j}}}$, ${\text{n}}_{{{\text{k}},{\text{j}}}}$ is the number of elements ${\text{x}}_{{\text{i}}}$ that belong to cluster ${\text{C}}_{{\text{j}}}$ labeled as ${\text{k}}$. To assess the overall performance of the clustering algorithm, the average Gini impurity index has been calculated:

3 $$G = \frac{1}{d}\mathop \sum \limits_{j = 1}^{d} G\left( j \right)$$

### Manual dataset cleaning

Manual dataset cleaning involved a thorough examination of all acquired microscopy images by different members of the research group. Cells for which we lacked high confidence about their identity were excluded using the Pandas DataFrame method .drop_duplicates. Exclusions were made for various reasons, including cells exhibiting rearrangements, slight mismatches in the focal plane between FLIM and immunofluorescence images, as well as notable changes in cell shape.

### Feature selection

Feature selection was implemented to enhance performance and model interpretability. After the initial training, the most important features were selected using the XGBoost built-in method .feature_importances_. These features were ordered from most to least important, and only the 116 most important features were used to train the highest-performing algorithm.

### Preprocessing

#### Missing values handling

Missing values, represented as NANs in the feature matrix, primarily originated from dark spots or cells lacking lipofuscin, particularly in autofluorescence intensity images: they were imputed with ‘zeros’ to retain their meaning using the Pandas DataFrame method .fillna(0), signifying the absence of NAD(P)H or lipofuscin in the acquired field.

#### Outliers handling

Outliers were detected using the scikit-learn LocalOutlierFactor^42^ function. Values identified as outliers were substituted with the average value of the feature using the scikit-learn SimpleImputer function.

#### Scaling

Values were scaled to improve model performance. Scaling was implemented using the scikit-learn MinMaxScaler function, which normalizes data within a range from the minimum to the maximum. Data would be 0 if equal to the minimum value and 1 if equal to the maximum; otherwise, they fell within the range [0,1].

#### Imbalanced dataset correction

The dataset was balanced in two ways. First, the parameter class_weight = 'balanced' was set during model initialization. Second, synthetic oversampling was implemented using the SMOTE class from the imbalanced learn library.

### Cross validation

Cross Validation was crucial to obtain a stable model. Given the imbalanced nature of the dataset, Cross Validation was implemented using the RepeatedStratifiedKFold function from the scikit-learn library. It divided the dataset into K parts and performed training for each dataset split, repeating the process a user-specified number of times. The stratified method preserved class proportions in each dataset fold. In this case, the dataset was divided into 5 folds and underwent 3 repetitions.

### Hyperparameters tuning

Hyperparameters tuning was performed to further enhance model performance, with grid search chosen as the optimization method. Grid search explored all possible hyperparameter configurations of the model, returning the model with the hyperparameters configuration that yielded the highest score. It was implemented using the scikit-learn function GridSearchCV. A detailed view of hyperparameters can be found in the provided code in the Supplementary Material.

### Performance evaluation

Four class-specific scores were calculated for performance evaluation: the area under an ROC curve, precision, recall, and accuracy. These scores were implemented using functions from scikit-learn, including roc_auc_score, precision_score, recall_score, and accuracy_score.

### Stability assessments

#### Tenfold cross validation

We increased the number of folds from 5 to 10, implementing tenfold repeated stratified cross-validation, using the RepeatedStratifiedKFold function of scikit-learn library. All training and Optuna-optimization steps were repeated in the same way as fivefold cross validation, but using 10 folds instead of 5.

#### Salzberg test

The ‘Salzberg test’^31^ is a method that involves shuffling the labels in the target vector of the training dataset, allowing the algorithm to learn from noise. The shuffling has been performed using numpy. Then, the model was trained and tested following the same protocol used for training with Optuna optimization.

#### Classification of excluded data

We attempted to classify data that were excluded from training during the dataset cleaning procedure. The Optuna-optimized XGBoost has been loaded from a saved file and used to classify the excluded data.

### Supplementary Information

Supplementary Information 1.

Supplementary Information 2.

## Data Availability

The dataset can be downloaded at 10.6084/m9.figshare.23765169.v1, and we recommend using the specific function implemented for import available at https://github.com/Biofaa/CellTypeClassification.
