# Supplementary material for: Machine-learning-guided recognition of α and β cells from label-free infrared micrographs of living human islets of Langerhans
Source: Sci Rep. 2024 Jun 20;14:14235. doi: 10.1038/s41598-024-65161-7 (PMC11190282; doi:10.1038/s41598-024-65161-7)
Supplement: Supplementary file 1 — Supplementary Information 1. [file 41598_2024_65161_MOESM1_ESM.docx]

**Supplementary Material**

**Title:**

**Machine-Learning-guided recognition of α and β cells from label-free infrared micrographs of living human islets of Langerhans**

Fabio Azzarello^a,*^, Francesco Carli^b^, Valentina De Lorenzi^a^, Marta Tesi^c^, Piero Marchetti^c^, Fabio Beltram^a^, Francesco Raimondi^b,*^, Francesco Cardarelli^a,*^

**Affiliations:**

^a^ NEST Laboratory - Scuola Normale Superiore, Piazza San Silvestro 12, Pisa, Italy.

^b^ Laboratorio di Biologia Bio@SNS, Scuola Normale Superiore, 56126, Pisa, Italy

^c^ Department of Clinical and Experimental Medicine, Islet Cell Laboratory, University of Pisa, Pisa, Italy.

* to whom correspondence should be addressed: fabio.azzarello@sns.it; francesco.raimondi@sns.it; francesco.cardarelli@sns.it


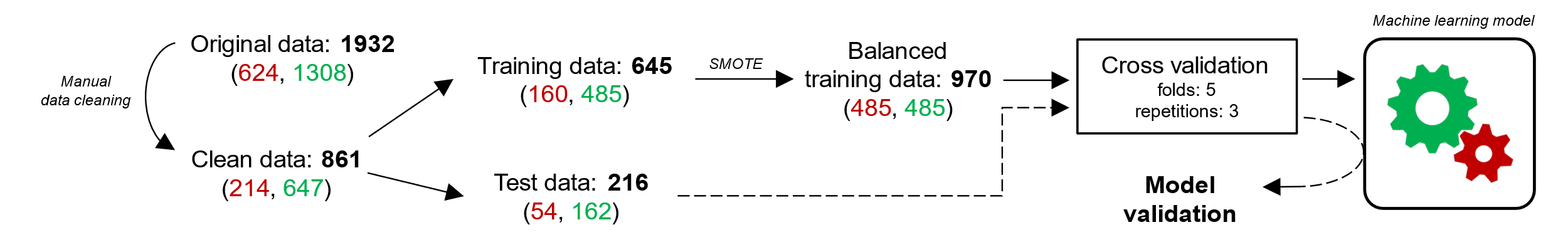


**Figure S1: Dataset Handling Through the Training and Testing Workflow.** The original dataset consists of a 1932x151 matrix, where the rows represent a single cell, and the columns are the computed features, with a total of 624 alpha cells (red) and 1308 beta cells (green). Before any analysis, the dataset has been cleaned by excluding cells with uncertain identity, which was done by manually cross-checking FLIM, autofluorescence, and immunofluorescence images. In total, 1071 cells were excluded from the analysis, leaving 861 cells. The dataset has been split into training and test sets with a 4:1 proportion to avoid overestimation of performance, resulting in 645 cells for training and 216 for testing. For optimal training, it is beneficial to have balanced classes, meaning the same number of alpha and beta cells. Class balance was achieved by generating synthetic data for alpha cells using the Synthetic Minority Oversampling Technique (SMOTE), which resulted in 970 cells in total with perfectly balanced classes (i.e., 485 cells for both alpha and beta cells). A repeated stratified 5-fold cross-validation was implemented for the training dataset, where the dataset is split into 5 folds, and each fold is used independently for training the algorithm. This process was repeated 3 times, resulting in 15 training scores. The final training score is the average of all the training scores. The testing set, which is an agnostic dataset used to test the model's performance, was treated in the same way, resulting in a final testing score used to validate the model's performance without overestimations.
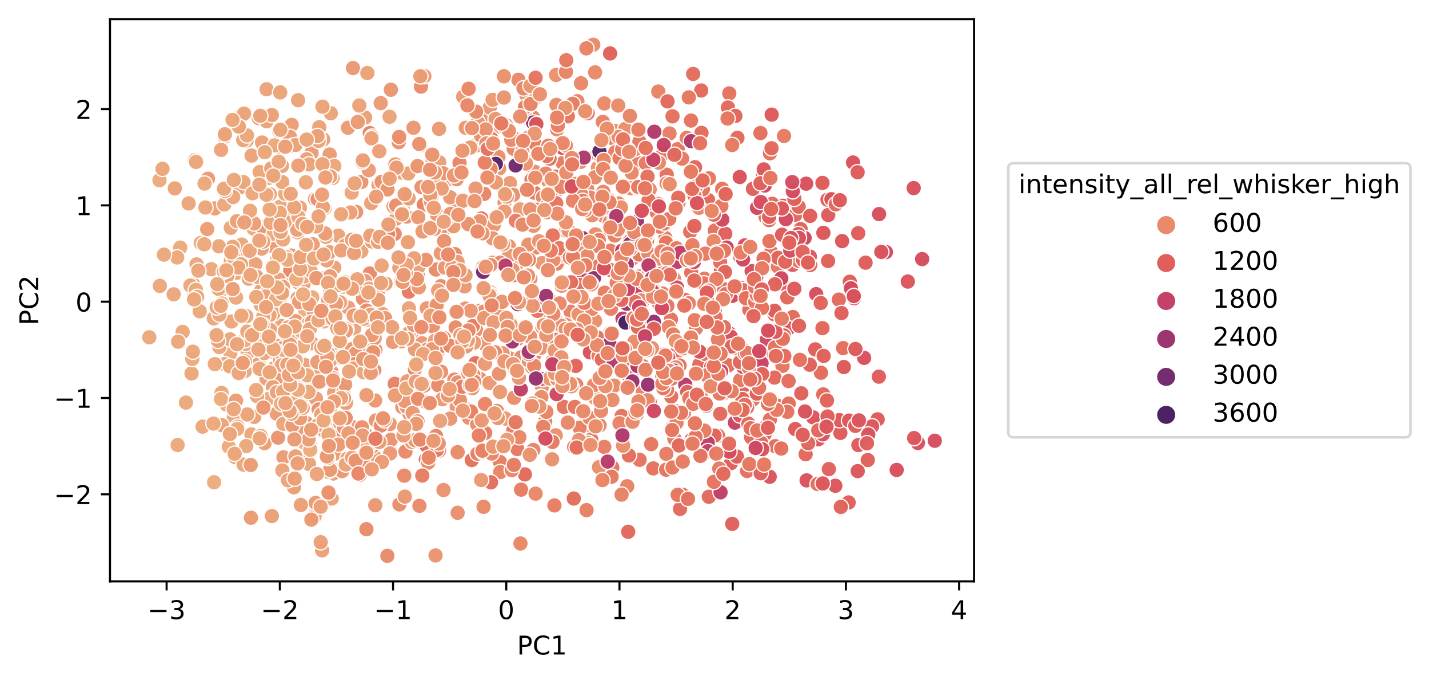


**Figure S2: Effect of the most important feature on XGBoost classification power.** After XGBoost optimization with Optuna, the most important features have been extracted using the XGboost *.feature_importances_* method. The most important feature was “intensity_all_whisker_high”, which is the higher whisker coordinate of a boxplot of the fluorescence intensity in the image. As evidenced by the colomap, the least intense cells are located on the left side of the plot and the most intense ones on the right side, following the cell type distribution of KDE plots in **Figure 4**.

|  | **Age** | **Sex** | **BMI** | **Diabetes (ND/T2D)** | **Cause of death** | **Insulin SI** |
| --- | --- | --- | --- | --- | --- | --- |
| **Donor 1** | 85 | M | 27.7 | ND | Cardiovascular disease | 1.37 |
| **Donor 2** | 80 | M | 23.03 | ND | Cardiovascular disease | 2.32 |
| **Donor 3** | 46 | M | 23.67 | ND | trauma | 3.9 |
| **Donor 4** | 79 | M | 26.81 | ND | Cardiovascular disease | 5.35 |

**Table S1: Relevant clinical information of Langerhans islet donors.** All the 4 donors were healthy, non-diabetic males. They were around 79-85 years, except one out of four. Since islets undergo autolysis fast after death, the only possible donors may be organ donors or brain-dead ones^1^. From 4 donors, 15 islets were extracted, ending with a total of 966 single cells extracted.

| **Islet ID** | **β-cells** | **α-cells** | **tot** | **β:α** | **β (%)** | **α (%)** |
| --- | --- | --- | --- | --- | --- | --- |
| Islet 1 | 94 | 8 | 102 | 11.8 | 92 | 8 |
| Islet 2 | 248 | 72 | 320 | 3.4 | 78 | 23 |
| Islet 3 | 76 | 56 | 132 | 1.4 | 58 | 42 |
| Islet 4 | 74 | 44 | 118 | 1.7 | 63 | 37 |
| Islet 5 | 68 | 38 | 106 | 1.8 | 64 | 36 |
| Islet 6 | 56 | 16 | 72 | 3.5 | 78 | 22 |
| Islet 7 | 44 | 56 | 100 | 0.8 | 44 | 56 |
| Islet 8 | 128 | 72 | 200 | 1.8 | 64 | 36 |
| Islet 9 | 98 | 86 | 184 | 1.1 | 53 | 47 |
| Islet 10 | 42 | 32 | 74 | 1.3 | 57 | 43 |
| Islet 11 | 60 | 54 | 114 | 1.1 | 53 | 47 |
| Islet 12 | 80 | 46 | 126 | 1.7 | 63 | 37 |
| Islet 13 | 88 | 8 | 96 | 11.0 | 92 | 8 |
| Islet 14 | 98 | 20 | 118 | 4.9 | 83 | 17 |
| Islet 15 | 54 | 16 | 70 | 3.4 | 77 | 23 |

**Table S2: cellularity of all measured Langerhans islets.** For each islet, the total number of β- and α- cells collected in a single microscopy frame is reported. The β:α cell ratio has been obtained by dividing the number of β-cells by the α- ones, while the relative amount of each cell type (i.e. β or α percentual) has been obtained by divinding the number of cells of one type (i.e. β or α) by the total number of cells in the islet (tot).


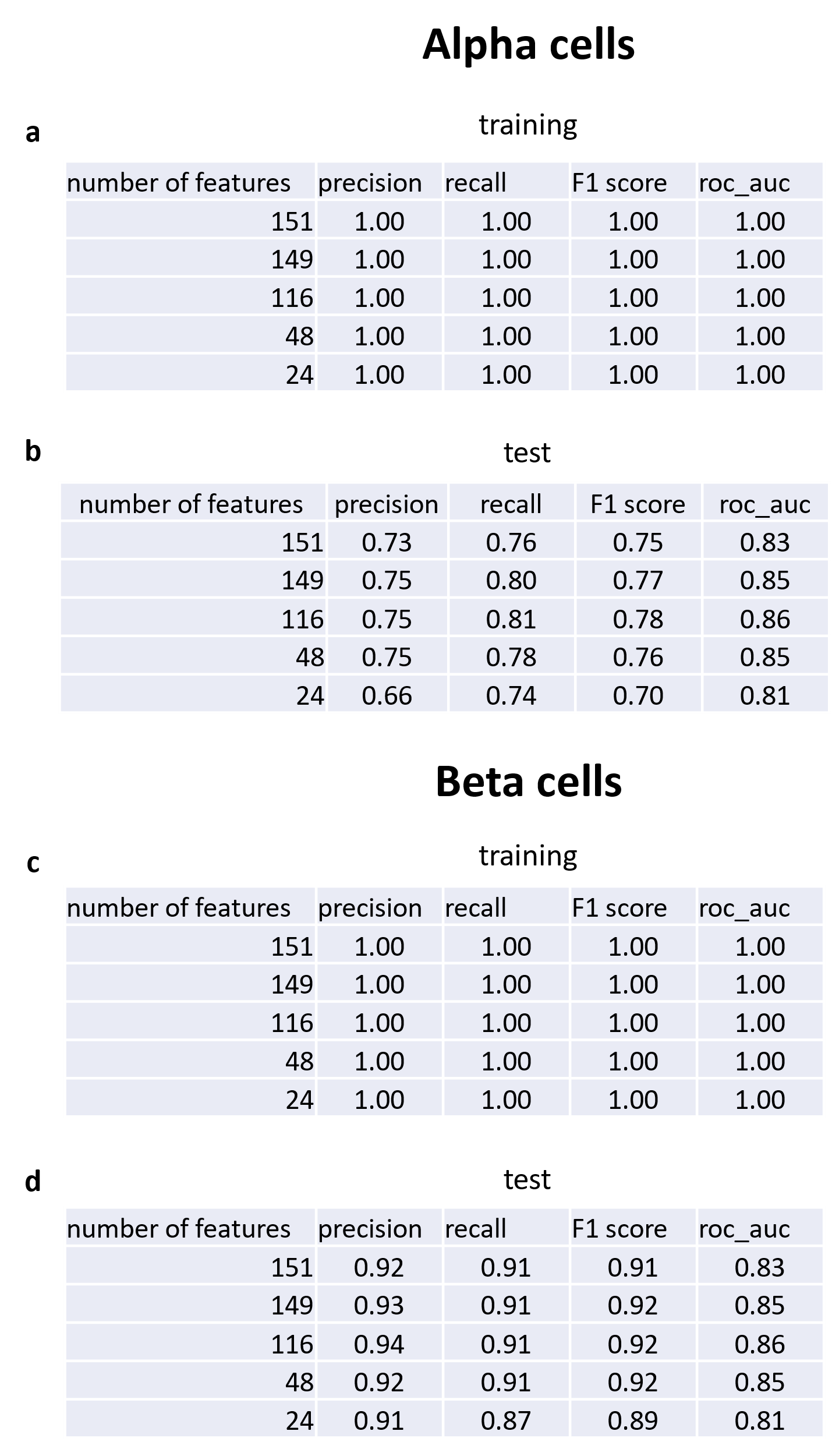


**Table S3: Optuna-Optimized Scores of XGBoost at Different Feature Selection Cutoff.** XGBoost has been trained and optimized using Optuna with varying feature selection cutoffs. **a)** For alpha cells during the training phase, the algorithm displayed the highest score of 1.0 for every computed metric. **b)** During the test phase, the highest-performing trial has been obtained using 116 features out of 151, with a ROC_AUC of 0.86. The remaining scores ranged from 0.75 to 0.81. Beta cells displayed similar results, but with higher scores. **c)** For beta cells during the training phase, the algorithm displayed a perfect score of 1.0 for every metric. **d)** During the test phase, the highest-performing trial was again achieved with the 116 most important features, resulting in a ROC_AUC of 0.86, with all scores higher than 0.90.


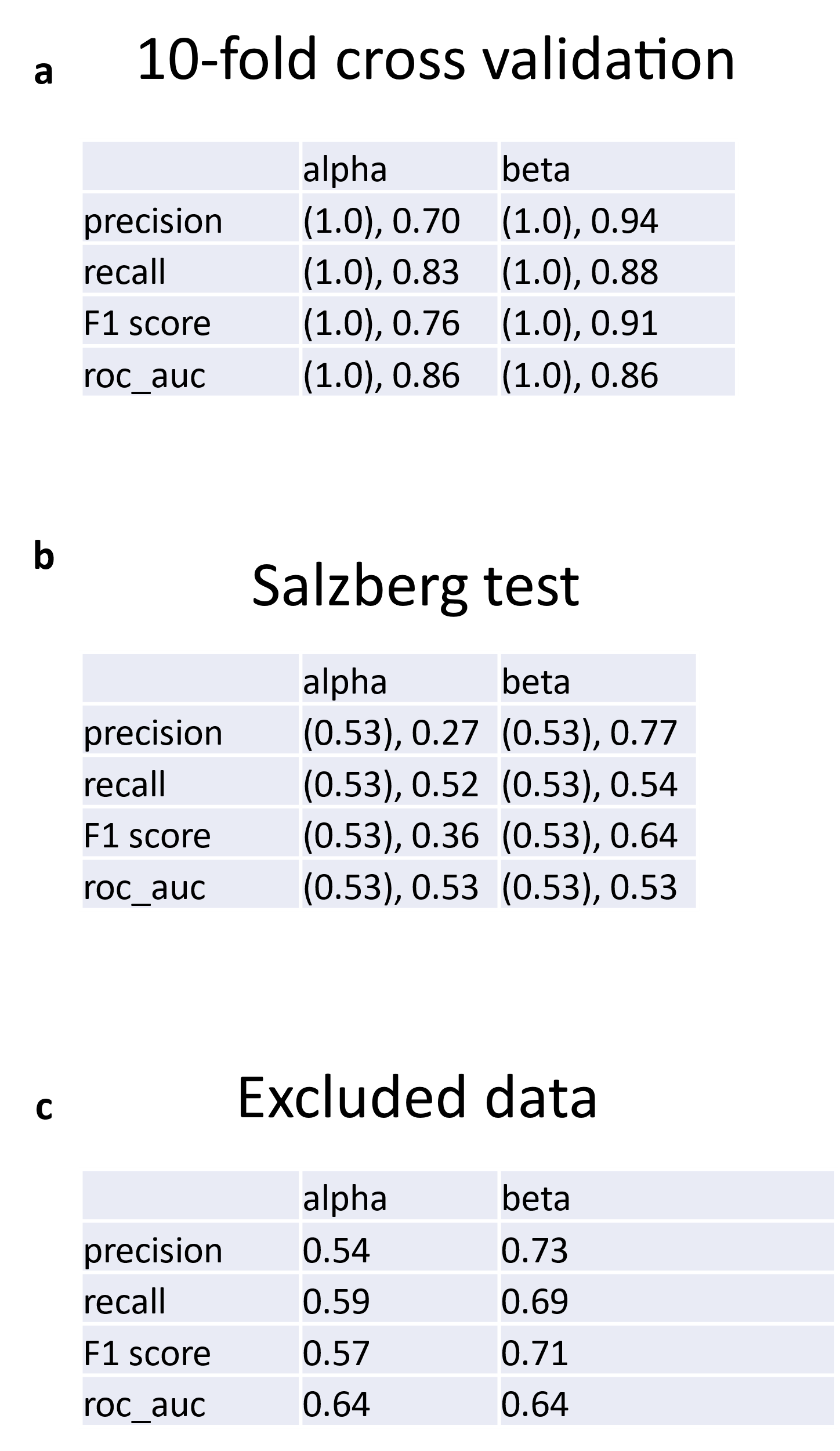


**Table S4: Assessment of Optuna-Optimized XGBoost Stability.** We performed various assessments to evaluate the stability of the Optuna-optimized XGBoost model. **a)** In a 10-fold repeated stratified cross-validation on the dataset, XGBoost displayed similar scores to the 5-fold cross-validation during the training phase, reinforcing the hypothesis that overfitting is absent. **b)** The Salzberg test involved shuffling the training target vector to make XGBoost learn from noise. The results showed a drop in both training and testing scores, as expected. This indicates that the original XGBoost was effectively learning from the data. **c)** In a final test, XGBoost was allowed to predict the outcomes for the 1071 out of 1932 cells that were discarded from the analysis. It displayed scores lower than those during the testing phase. This could be a clue of either overfitting or erroneous manual labeling. Based on the previous stability assessments, it is more likely that the latter hypothesis is accurate.
